# The Diagnostic Accuracy of Methylmalonic Acid to Detect Inadequate Vitamin B-12 Status Relative to 3cB-12 Was Higher Compared with That of Total Cobalamin and Total Homocysteine in a Cross-Sectional Survey of Apparently Healthy US Adults^☆^

**DOI:** 10.1016/j.cdnut.2026.107704

**Published:** 2026-04-22

**Authors:** Ekaterina M Mineva, Maya R Sternberg, Christine M Pfeiffer

**Affiliations:** Division of Laboratory Sciences, Centers for Disease Control and Prevention (CDC), National Center for Environmental Health, Atlanta, GA, United States

**Keywords:** MMA, tHcy, cobalamin, NHANES, prevalence, diagnostic accuracy

## Abstract

**Background:**

Diagnosing low vitamin B-12 status is important to reduce megaloblastic anemia and neurological disorders. The lack of a highly specific and sensitive clinical marker and questions about appropriate cutoffs hamper early detection.

**Objectives:**

We evaluated the predictive power of 3 conventional markers for inadequate vitamin B-12 status [total cobalamin (B-12), methylmalonic acid (MMA), and total homocysteine (tHcy)] using the combined vitamin B-12 indicator, 3cB-12 (≤**−**0.5), as a reference. Additionally, we derived optimum cutoffs for each conventional marker and compared the prevalence with that from traditional cutoffs.

**Methods:**

Using NHANES 1999–2004 data for vitamin B-12, MMA, and tHcy for persons ≥20 y with normal renal function, we devised a training set (1999–2002, *n* = 7469) to derive new optimum cutoffs and a validation set (2003–2004, *n* = 3641) to test them. All analyses were weighted and accounted for clustered design.

**Results:**

In the training set, the area under the curve for MMA was 0.963, followed by B-12 (0.944) and tHcy (0.937); it further increased with 2- and 3-marker models (0.985–0.998). In the validation set, the diagnostic accuracy (Youden’s index) for the new optimum cutoffs was 0.80 for MMA >229 nmol/L and 0.69 for vitamin B-12 <220 pmol/L and tHcy >10.4 μmol/L. Among the traditional cutoffs, Youden’s index varied (0.30–0.79); vitamin B-12 <126 or <148 pmol/L and MMA >376 nmol/L had the highest specificity (>99%). The latter also had reasonable sensitivity (54.9%) and generated an apparent (2.35%) and a sensitivity/specificity-adjusted prevalence (2.48%) close to the 3cB-12 reference estimate (2.74%).

**Conclusions:**

When using a single marker, MMA >376 nmol/L may be a useful cutoff for healthy populations with low (<5%) prevalence of inadequate vitamin B-12 status to minimize false positives, whereas MMA >229 nmol/L may be preferred for screening higher-risk groups to detect more cases.

## Introduction

Vitamin B-12 is essential for DNA and methionine synthesis, which affects red blood cell formation, as well as brain and nerve functioning [1]. If left untreated, it can lead to megaloblastic anemia and neurological or cognitive disorders, some of which may become permanent [[1], [2], [3]]. Although the prevalence of vitamin B-12 deficiency is low in the US population [4,5], it can still affect a large number of people, especially elderly Americans. Thus, early diagnosis is not only clinically important but also of public health interest. However, detecting low vitamin B-12 status is complicated by subtle clinical manifestations, symptoms overlapping with those of other conditions, the lack of a reliable clinical marker, and the absence of scientific consensus on diagnostic cutoff points.

There are 4 generally accepted blood-based biomarkers for vitamin B-12 status: serum vitamin B-12 or total cobalamin (B-12), methylmalonic acid (MMA), total homocysteine (tHcy), and the transport protein holotranscobalamin (holoTC), each used with multiple cutoff points [5]. Although elevated tHcy is not specific for vitamin B-12 status, elevated MMA is a specific marker of vitamin B-12 function and is considered the gold standard for diagnosing deficiency; however, it is not commonly used in the clinical setting [1,6]. B-12 measurements are widely available, inexpensive, and therefore are most commonly used in the clinical setting as well as at the population level [5]. HoloTC measures the biologically active B-12 but is rarely used outside the research setting. Non-nutritional factors, such as renal impairment, genetic factors, and aging, can also influence these biomarkers, particularly MMA and tHcy [1,6].

To overcome inadequate sensitivity and/or specificity of single markers, the concurrent or sequential use of 2 markers, typically B-12 and MMA, has been considered as more reliable both diagnostically and epidemiologically [2,[6], [7], [8], [9], [10]]. Furthermore, Fedosov et al. [11,12] developed a combined index for vitamin B-12 status, named combined indicator of vitamin B-12 status (cB-12), which integrates some or all of the 4 markers along with the individual’s age. This indicator is reported to be the most comprehensive diagnostic tool for vitamin B-12 status, more reliable than any single marker [11,12]. An earlier analysis using data from the NHANES indicated that single markers overestimated the prevalence of inadequate vitamin B-12 status when compared with 3cB-12 (B-12, MMA, and tHcy) [4].

Several studies have used cB-12 as a reference to evaluate the diagnostic accuracy of conventional, single markers for detecting vitamin B-12 deficiency across healthy and diseased populations [9,10,[13], [14], [15]]. However, to our knowledge, the diagnostic accuracy of these conventional markers compared with cB-12 has not yet been investigated in apparently healthy US adults to identify the extent of inadequate vitamin B-12 status. Our objectives were to *1*) use the NHANES data for B-12, MMA, and tHcy (no holoTC data available) to determine the predictive power of conventional markers, singly and in combination, relative to 3cB-12 as a reference point for inadequate vitamin B-12 status (≤–0.5); *2*) establish new optimum cutoffs for each conventional marker by using a portion of the dataset (“training set”) and then evaluate the diagnostic accuracy of the new cutoffs using the remainder of the dataset (“validation set”); and *3*) using the validation set, representative of the US population, compare the prevalence of vitamin B-12 inadequacy using both the newly established optimum cutoffs and commonly used traditional cutoffs with the reference prevalence obtained with 3cB-12. Although our main analysis focused on US adults with normal renal function to allow for clear interpretation, we carried out a subanalysis in US adults regardless of their renal function to assess differences in findings because sometimes investigators cannot limit their study group to persons with normal renal function.

## Methods

### Participants and survey design

Since 1999, NHANES is a cross-sectional, continuous survey designed to assess the health and nutritional status of the US noninstitutionalized, civilian population [16]. Data are publicly released in 2-y cycles. Conducted by the National Center for Health Statistics (NCHS), Centers for Disease Control and Prevention (CDC), the survey is nationally representative and uses a complex, stratified, multistage probability sample. Participants first undergo a detailed home interview, which includes self-reported demographic, socioeconomic, dietary, and health-related questions. They are then invited to visit a mobile examination center (MEC), where a series of health examinations are performed and biological specimens are collected. Interview and examination response rates for each survey cycle are publicly available and fluctuated ∼80% for the 3 cycles (1999−2000, 2001−2002, and 2003−2004) that analyzed all 3 markers (B-12, MMA, and tHcy) [17]. Participants >18 y consented. The NCHS Ethics Review Board approved NHANES [18].

### Biomarker measurements

CDC’s Nutritional Biomarkers Laboratory analyzed serum B-12 by the Bio-Rad Quantaphase II radioassay, plasma MMA by GC-MS, and plasma tHcy by the Abbott Laboratories fluorescence polarization immunoassay. Long-term (1999−2004) assay imprecision coefficients of variation were mostly ≤5%. Detailed laboratory method information is provided on the NHANES website separately for each survey cycle [19].

### Study variables

We used renal function as a variable because its impairment increases MMA and tHcy concentrations independent of vitamin B-12 status [[20], [21], [22]] and has also been shown to affect 3cB-12 [4]. Renal function was determined by calculating the estimated glomerular filtration rate (eGFR) using the Chronic Kidney Disease Epidemiology Collaboration (CKD-EPI) creatinine equation, without considering race [23]. To reduce the potential impact of early renal disease, we extended the CKD-EPI eGFR classification based on the presence or absence of albuminuria according to the National Kidney Foundation classification system [24], defining normal renal function as an eGFR ≥60 mL/(min × 1.73 m^2^) and the absence of albuminuria.

### Statistical analysis

Statistical analyses were carried out using SAS for Windows software version 9.4 (SAS Institute) and SAS callable SUDAAN software version 11 (RTI) to account for the complex survey design. We performed the research following a prespecified data analysis plan that was approved by all authors before conducting the statistical analysis. The combined indicator of vitamin B-12 status, 4cB-12_,_ is calculated as follows: cB-12 = log_10_ [(holoTC × B-12) / (MMA × tHcy)] − (age factor) [11]. We used a modified equation to calculate 3cB-12 after accounting for the missing marker holoTC [12]. No folate correction was used because the median serum folate concentrations in US adults based on postfortification data from NHANES 1999–2004 were >10 nmol/L because of the mandatory folic acid fortification introduced in 1996 [25]. To define vitamin B-12 status, we combined data from the proposed epidemiological low and transitional cB-12 cutoffs and referred to it as “inadequate” (cB-12 ≤−0.5), whereas adequate vitamin B-12 status was defined as cB-12 >−0.5.

The analysis was based on 3 NHANES cycles (1999–2000, 2001–2002, and 2003–2004). To ensure we did not use the same data twice during evaluation of the new optimum cutoffs, we employed a training dataset (1999−2002) to independently estimate these values, as well as their sensitivity and specificity. This allowed us to adjust the prevalence of inadequate vitamin B-12 status in the validation dataset (2003−2004) using the sensitivity and specificity of each optimum cutoff. Four-year MEC survey weights provided in the demographic dataset were used for the 1999−2002 training dataset, whereas 2-y MEC weights were used for NHANES 2003–2004.

For our main analysis, we limited our dataset to participants who had data for all 3 markers B-12, MMA, and tHcy (*n* = 15,853 participants in the training dataset). We excluded data from anyone who was <20 y (*n* = 7012), pregnant and/or lactating females (*n* = 586), and participants ≥20 y with abnormal renal function (*n* = 786). Our final analytical sample for participants ≥20 y with normal renal function consisted of 7469 persons for the training dataset (Supplementary Figure 1). The validation dataset consisted of 3641 adults ≥20 y after the same exclusions. There were 157 and 98 adult participants who had inadequate vitamin B-12 status in the training and validation dataset, respectively. Missing laboratory measurements, including those used to calculate eGFR, primarily reflect specimen availability and laboratory processing constraints, and differences in laboratory sample sizes are common across NHANES components. Eligibility variables (e.g., pregnancy or lactation status) were subject to NHANES skip patterns; therefore, exclusions were applied only when eligibility criteria were explicitly met, and analyses were restricted to participants with complete data for all variables used, acknowledging potential residual uncertainty in eligibility classification. We also carried out a subanalysis in US adults regardless of their renal function where we did not exclude participants with abnormal renal function. We assessed diagnostic accuracy using AUC for receiver operating characteristic (ROC) models and evaluated individual cutoffs using sensitivity, specificity, and Youden’s index (sensitivity + specificity − 1).

#### Diagnostic accuracy of different biomarker models

We used weighted ROC curves to assess the AUC for detecting inadequate vitamin B-12 status (3cB-12 ≤−0.5) across different logistic regression models that incorporated both single and multiple conventional markers (B-12, MMA, and tHcy) using the training dataset. Statistically significant difference in the AUC between single and combinations of multiple markers was based on a *P* value < 0.05. The 95% percentile bootstrap confidence intervals (CIs) and *P* values comparing AUCs were based on resampling 2000 bootstrap samples [26]. The bootstrapping algorithm was implemented using SAS survey procedures that allows for replicate weights [27].

#### Optimum biomarker cutoffs for detecting inadequate vitamin B-12 status

We used the training dataset to determine the optimum cutoff for each conventional marker and its respective sensitivity and specificity in predicting inadequate vitamin B-12 status referenced to 3cB-12 ≤–0.5 for adults with normal renal function and for all adults separately. The optimum cutoff was identified as the point that is closest in distance to a perfect test, which sits at the upper left-hand corner of the ROC plot where the sensitivity is 100% and 1 − specificity is 0% (i.e., specificity is 100%). The CIs for optimum cutoffs were derived from a similar bootstrapping procedure described earlier. We then determined the sensitivity and specificity at each optimum cutoff as weighted percentages. The specificity indicates the likelihood of being accurately categorized as having adequate vitamin B-12 status. The SEs for the 95% CI for sensitivity and specificity were calculated using Taylor series linearization based on a logit transformation. Finally, Youden’s index, an indicator of diagnostic accuracy [28], was calculated by summing the sensitivity and specificity and subtracting 1.

#### Prevalence for traditional and new optimum cutoffs for detecting inadequate B-12 status

Using the validation dataset, we estimated both the apparent and adjusted prevalence, sensitivity, specificity, and Youden’s index. This evaluation was performed for the new optimum cutoffs as well as for selected traditional cutoffs as follows: B-12 <148 pmol/L (low B-12) and <223 pmol/L (inadequate B-12) [29]; B-12 <126 pmol/L (high risk of B-12 deficiency) and <287 pmol/L (not optimal B-12 status) [30]; MMA >271 nmol/L and >376 nmol/L [31], and age-specific MMA (>250 nmol/L for 20–39 y, >290 nmol/L for 40–69 y, and >320 nmol/L for ≥70 y) [20]; and tHcy >13 μmol/L [32]. The apparent prevalence, evaluated in the validation dataset, was adjusted using the sensitivity and specificity of the optimum cutoffs estimated from the training dataset and denoted as adjusted prevalence. The 95% CI was calculated using Taylor’s series linearization based on a logit transformation. The sensitivity, specificity, and Youden’s index of all cutoffs were calculated as described in the previous section. Because 3cB-12 was used as a reference, its sensitivity and specificity were designated as 100%.

## Results

### Predictive power of single or multiple conventional markers (training set)

**FIGURE 1:**
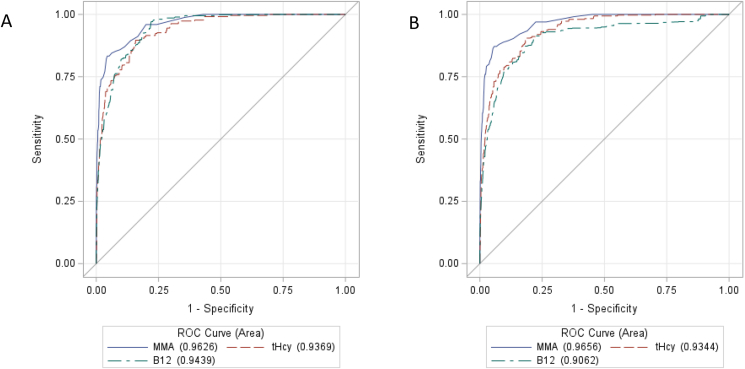
Receiver operating characteristic (ROC) curves for the 3 conventional markers predicting inadequate (low or transitional) vitamin B-12 status (3cB-12 ≤−0.5) in US adults aged ≥20 y with normal renal function (A) and in all adults (B), NHANES 1999−2002. The 3cB-12 score was calculated using the conventional markers B-12, MMA, and tHcy. Sample size was 7469 for adults with normal renal function and 8255 for all adults. B-12, serum vitamin B-12; cB-12_,_ combined indicator of vitamin B-12 status; MMA, plasma methylmalonic acid; tHcy, plasma total homocysteine.

Of all single markers, MMA had the highest predictive power for identifying inadequate vitamin B-12 status (i.e., 3cB-12 ≤−0.5) in adults with normal renal function with an AUC of 0.963, followed by B-12 with 0.944 and tHcy with 0.937 (Table 1, Figure 1A). A pairwise comparison of the AUC revealed that MMA had significantly higher diagnostic accuracy than B-12. The diagnostic accuracy, assessed by AUC, improved for the 2-marker models, with higher AUCs when MMA was included (AUC >0.985). As expected, the combination of all 3 markers had the highest diagnostic accuracy (AUC of 0.998) but the improvement in AUC from the 2-marker models was only marginal. Lastly, MMA also showed the highest predictive power in all adults regardless of renal function with an AUC of 0.966, followed by tHcy with 0.934 and B-12 with 0.906 (Supplementary Table 1, Figure 1B).

**TABLE 1:** AUC of the ROC to detect inadequate (low or transitional) vitamin B-12 status (3cB-12 ≤–0.5) for various models of single or combined conventional markers in US adults aged ≥20 y with normal renal function, NHANES 1999−2002^1^

Biomarker models	Weighted AUC (95% CI)^2^
Single marker	
B-12	0.944^a^ (0.930, 0.958)
MMA	0.963^b^ (0.942, 0.982)
tHcy	0.937^a,b^ (0.919, 0.954)
Multiple markers	
B-12, MMA	0.985^c,d^ (0.974, 0.995)
B-12, tHcy	0.977^b,d^ (0.972, 0.982)
MMA, tHcy	0.993^b,c^ (0.990, 0.996)
MMA, tHcy, B-12	0.998^e^ (0.997, 0.999)

Abbreviations: B-12, serum vitamin B-12; cB-12_,_ combined indicator of vitamin B-12 status; CI, confidence interval; CKD-EPI, Chronic Kidney Disease Epidemiology Collaboration, eGFR, estimated glomerular filtration rate; MMA, plasma methylmalonic acid; ROC, receiver operating characteristic; tHcy, plasma total homocysteine.

1 Pregnant and lactating females were excluded; only participants ≥20 y with normal renal function and complete biomarker data were included. Sample size was 7469 participants, of whom 157 were categorized as having inadequate vitamin B-12 status. Normal renal function was defined as an eGFR ≥60 [mL/(min × 1.73 m^2^)] using the CKD-EPI eGFR calculation without considering race.

2 Groups with different superscript letters are significantly different from each other based on pairwise testing (*P* < 0.05). Bootstrap 95% CIs.

### ROC derived optimum cutoffs for conventional markers and their diagnostic accuracy (training set)

On the basis of data from adults with normal renal function in NHANES 1999−2002, the new MMA optimum cutoff of >229 nmol/L showed the highest specificity (92.3%) and diagnostic accuracy (Youden’s index: 0.77) among the 3 markers, indicating the highest diagnostic performance in distinguishing between adults with inadequate (3cB-12 ≤−0.5) and adequate (3cB-12 >−0.5) vitamin B-12 status (Table 2). The new tHcy optimum cutoff of >10.4 μmol/L showed the highest sensitivity (89.6%) but lowest specificity (83.5%). The new optimum cutoff for B-12 (<220 pmol/L) and for tHcy showed similar diagnostic accuracy (Youden’s index: 0.72 and 0.73, respectively).

**TABLE 2:** ROC derived optimum cutoffs and corresponding diagnostic performance to detect inadequate (low or transitional) vitamin B-12 status (3cB-12 ≤–0.5) based on single conventional markers in US adults aged ≥20 y with normal renal function, NHANES 1999−2002^1^

Biomarker	Optimum cutoff (95% CI)^2^	Sensitivity, % (95% CI)^3^	Specificity, % (95% CI)^3^	Youden’s index^4^
B-12	220 (203, 240) pmol/L	85.6 (76.7, 91.5)	86.6 (85.8, 87.3)	0.72
MMA	229 (180, 270) nmol/L	85.2 (73.3, 92.4)	92.3 (91.5, 93.0)	0.77
tHcy	10.4 (10.3, 11.4) μmol/L	89.6 (85.5, 93.2)	83.5 (82.3, 84.6)	0.73

Abbreviations: B-12, serum vitamin B-12; cB-12_,_ combined indicator of vitamin B-12 status; CI, confidence interval; CKD-EPI, Chronic Kidney Disease Epidemiology Collaboration, eGFR, estimated glomerular filtration rate; MMA, plasma methylmalonic acid; ROC, receiver operating characteristic; tHcy, plasma total homocysteine.

1 Pregnant and lactating females were excluded; only participants ≥20 y with normal renal function and complete biomarker data were included. Sample size was 7469 participants, of whom 157 were categorized as having inadequate vitamin B-12 status. Normal renal function was defined as an eGFR ≥60 [mL/(min × 1.73 m^2^)] using the CKD-EPI eGFR calculation without considering race.

2 Optimum cutoff points for the conventional markers correspond to the value with the minimum distance to the (0,1) point on the ROC plot (optimized sensitivity and specificity). CIs for the optimal cutoff points were derived from bootstrap 95% CIs.

3 Standard errors were estimated using Taylor series linearization. Confidence intervals for sensitivity and specificity were calculated using a logit transformation.

4 Youden’s index was calculated as sensitivity + specificity − 1.

We observed the same pattern for optimum cutoffs for all adults regardless of their renal function in NHANES 1999−2002: MMA >259 nmol/L had the highest specificity (94.2%) and diagnostic accuracy (Youden’s index: 0.81); tHcy >10.4 μmol/L had the highest sensitivity (90.5%); and the sensitivity and specificity for B-12 <240 pmol/L was intermediate (Supplementary Table 2).

### Prevalence of inadequate vitamin B-12 status by optimum or traditional cutoffs and their diagnostic accuracy (validation set)

The new optimum cutoffs for adults with normal renal function, estimated in the training dataset and tested in the validation dataset, provided a higher apparent prevalence (9.81%–20.5%) compared with the 3cB-12 reference estimate (2.74%) (Table 3). MMA >229 nmol/L had the highest Youden’s index (0.8) among the 3 optimum cutoffs and the traditional cutoffs. MMA >229 nmol/L also had a comparable adjusted prevalence (2.70%) with the 3cB-12 estimate. Among the traditional cutoffs, B-12 <223 pmol/L had the highest Youden’s index (0.79) but it underestimated the prevalence (1.16%) when compared with 3cB-12. MMA >376 nmol/L, B-12 <126 pmol/L, and MMA >age-specific cutoff generated adjusted prevalences (2.48%, 2.45%, and 2.43%, respectively) closest to the 3cB-12 estimate but had lower Youden’s indices (0.54, 0.30, and 0.71, respectively). MMA >376 nmol/L showed the smallest difference between the apparent (2.35%) and adjusted prevalence (2.48%), a high specificity (99.1%), and a reasonably high sensitivity (54.9%).

**TABLE 3:** Diagnostic performance and weighted apparent and adjusted prevalence for optimum and traditional cutoffs for US adults aged ≥20 y with normal renal function, NHANES 2003–2004^1^

Biomarker categories (reference for cutoffs)	Sensitivity, % (95% CI)^2^	Specificity, % (95% CI)^2^	Youden’s index^3^	Apparent prevalence^4^ (%) (95% CI)^2^	Adjusted prevalence^4^ (%) (95% CI)^2^
3cB-12^5^ ≤−0.5 [12]	100	100	—	2.74 (1.94, 3.85)	2.74 (1.94, 3.85)
Optimum cutoffs					
B-12 <220 pmol/L	90.6 (84.7, 94.4)	88.0 (84.7, 90.7)	0.69	14.1 (11.1, 17.8)	0.96 (0.01, 57.1)
MMA >229 nmol/L	87.9 (78.4, 93.5)	92.4 (90.5, 93.9)	0.80	9.81 (7.90, 12.1)	2.70 (0.91, 7.75)
tHcy >10.4 μmol/L	88.1 (74.7, 94.9)	81.4 (78.7, 83.8)	0.69	20.5 (17.8, 23.5)	5.44 (2.47, 11.6)
Traditional cutoffs					
B-12 <148 pmol/L [29]	41.9 (33.9, 50.5)	99.2 (98.5, 99.5)	0.41	1.97 (1.37, 2.84)	1.27 (0.29, 5.32)
B-12 <223 pmol/L [29]	91.7 (85.4, 95.5)	87.3 (83.7, 90.2)	0.79	14.9 (11.7, 18.7)	1.16 (0.01, 48.5)
B-12 <126 pmol/L [30]	30.6 (19.4, 44.8)	99.6 (99.2, 99.8)	0.30	1.25 (0.86, 1.80)	2.45 (1.10, 5.38)
B-12 <287 pmol/L [30]	96.4 (89.6, 98.8)	67.2 (63.9, 70.4)	0.64	34.5 (31.2, 38.0)	2.32 (0.20, 22.2)
MMA >376 nmol/L [31]	54.9 (40.9, 68.1)	99.1 (98.6, 99.4)	0.54	2.35 (1.84, 3.01)	2.48 (1.44, 4.25)
MMA >271 nmol/L [31]	79.8 (69.8, 87.1)	96.1 (94.8, 97.1)	0.76	6.00 (4.73, 7.59)	3.47 (1.88, 6.31)
MMA > age-specific cutoff [20]	73.9 (59.7, 84.3)	96.9 (95.8, 97.7)	0.71	5.04 (3.91, 6.46)	2.43 (1.07, 5.41)
tHcy >13 μmol/L [32]	71.3 (59.2, 80.9)	95.2 (93.9, 96.2)	0.66	6.66 (5.24, 8.28)	3.92 (2.05, 7.41)

Abbreviations: B-12, serum vitamin B-12; CI, confidence interval; cB-12_,_ combined indicator of vitamin B-12 status; CKD-EPI, Chronic Kidney Disease Epidemiology Collaboration, eGFR, estimated glomerular filtration rate; MMA, plasma methylmalonic acid; tHcy, plasma total homocysteine.

1 Pregnant and lactating females were excluded; only participants ≥20 y with normal renal function and complete biomarker data were included. Sample size was 3641 participants, of whom 98 were categorized as having inadequate vitamin B-12 status. Normal renal function was defined as an eGFR ≥60 using the CKD-EPI eGFR calculation without considering race.

2 Standard errors were estimated using Taylor series linearization. Confidence intervals for sensitivity, specificity, and prevalence were calculated using a logit transformation.

3 Youden’s index was calculated as sensitivity + specificity − 1.

4 Apparent prevalence was adjusted using the sensitivity and specificity of the traditional cutoff points estimated from NHANES 1999–2002.

5 3cB-12 was considered the gold standard with 100% sensitivity and 100% specificity.

We observed similar patterns in the validation set for all adults regardless of renal function; here, the reference prevalence of inadequate B-12 status (3cB-12 ≤ −0.5) was slightly higher (3.23%) (Supplementary Table 3). MMA >259 nmol/L had the highest Youden’s index (0.79) among the 3 optimum cutoffs and the traditional cutoffs. The new optimum cutoffs for B-12 and tHcy had not only lower Youden’s indices but also different apparent and adjusted prevalences compared with 3cB-12. Among the traditional cutoffs, MMA >271 nmol/L had the highest Youden’s index (0.77) but it overestimated the prevalence (3.81%) when compared with 3cB-12. B-12 <126 pmol/L provided the closest adjusted prevalence (3.11%) to 3cB-12 but it had the lowest Youden’s index (0.27). MMA >376 nmol/L showed again the smallest difference between the apparent (3.13%) and adjusted prevalence (2.80%), a high specificity (98.8%), and a reasonably high sensitivity (60.1%).

## Discussion

We used data from a cross-sectional, nationally representative survey to investigate the utility of 3 conventional markers—B-12, MMA, and tHcy—to assess inadequate vitamin B-12 status in apparently healthy US adults relative to 3cB-12 as a reference. To simplify interpretation, we focused our main analysis on adults with normal renal function. MMA, either as a single marker or in combination with a second marker, had the highest predictive power. Furthermore, the newly derived optimum cutoff for MMA (>229 nmol/L) had the highest diagnostic accuracy (AUC and Youden’s index) compared with newly derived optimum cutoffs for B-12 or tHcy and with traditional cutoffs.

Multiple markers of vitamin B-12 status, either singly or combined, are generally considered to improve clinical diagnosis and epidemiologic interpretation [2,[6], [7], [8],^33^,^34^], albeit individual tests can lead to contradictory indications and/or widely different population prevalences depending on the marker and/or cutoff used [35,36]. Nonetheless, practical challenges such as cost and test accessibility present barriers for the routine use of multiple markers. We therefore asked the question of which single, conventional marker had the highest predictive power and diagnostic accuracy relative to 3cB-12 as a reference. Our analysis showed that it was MMA. Multiple reviews support our finding, emphasizing that MMA is the most sensitive and specific marker of vitamin B-12 status for individuals of all ages with normal renal function [6,34]. Naturally, combining MMA with either B-12 or tHcy or with both improved its predictive power even further. Others have also noted that the combination of B-12 and MMA is particularly useful in daily practice and research settings [7,8]. Earlier findings by Fedosov using clinical data from 3 populations indicated that MMA (0.916) had nearly as high predictive power (AUC) for vitamin B-12 deficiency as holoTC (0.928) and was superior to B-12 (0.881) and tHcy (0.872) [37]. Using data from a large Swiss retrospective patient cohort, Campos et al. [15] also found a slightly higher AUC for holoTC (0.92) compared with MMA (0.91), B-12 (0.9), and tHcy (0.78). However, the authors concluded that in females <50 y and in males, holoTC, MMA, or tHcy did not appear superior to B-12 for the detection of B-12 deficiency, whereas in females ≥50 y, holoTC seemed to be the preferred first-line marker, pointing to age and sex differences in the diagnostic accuracy of B-12 biomarkers.

Campos et al. [15] also determined optimum cutoffs for detecting subclinical B-12 deficiency (4cB-12 ≤−0.5 and >−1.5); their new cutoffs were similar to ours for B-12 (<229 compared with <220 pmol/L) and MMA (>245 compared with >229 nmol/L), especially when considering that we used a slightly different definition (3cB-12 ≤−0.5); however, their cutoff for tHcy was higher (>15 compared with >10.4 μmol/L). Because our study had far fewer participants with “inadequate” B-12 status (157 and 98 adults with 3cB-12 ≤−0.5 in the training and validation dataset, respectively) compared with the Swiss study (1025 patients with low B-12, possible B-12 deficiency, and probable B-12 deficiency), we had to combine data from the low and transitional cB-12 cutoffs into 1 group (3cB-12 ≤−0.5). This may have led to higher sensitivity at the new optimum cutoffs in our study compared with the Swiss study.

Even though MMA >229 nmol/L had the highest Youden’s index for distinguishing between inadequate and adequate B-12 status in our analysis (0.8), the apparent prevalence was higher (9.81%) than the adjusted (2.70%) or the reference prevalence based on 3cB-12 (2.74%). On the other hand, MMA >376 nmol/L had a lower Youden’s index (0.54) but similar apparent (2.35%) and adjusted (2.48%) prevalences that were also close to the reference prevalence. When the disease prevalence is low (<5%), even tests with high sensitivity and specificity produce a fair number of false positives, unless the specificity is very high. Although MMA >229 nmol/L detects more positive cases overall, it also yields a higher number of false positives, which leads to an overestimation of the apparent prevalence. Using 3cB-12 as a gold standard, the positive predictive value for MMA >229 nmol/L is lower (∼20% at a specificity of 92.4%) than for MMA >376 nmol/L (∼60% at a specificity of 99.1%). Thus, from a standpoint of detecting true cases, such as in a presumably healthy population, MMA >376 nmol/L is superior. On the other hand, MMA >229 nmol/L may be preferable if one tries to rule out cases because it has a higher negative predictive value or if the objective is to screen high-risk groups (e.g., older persons or populations with a higher prevalence of B-12 deficiency) because sensitivity matters more when the priority is not to miss deficiency. The behavior of the traditional B-12 cutoff of <148 pmol/L compared with the new B-12 cutoff of <220 pmol/L mirrors what we described above for MMA >376 nmol/L compared with >229 nmol/L. The traditional B_12_ cutoff had a very high specificity (99.2%; sensitivity of 41.0%) and similar apparent (1.97%) and adjusted prevalence (1.27%) close to the reference prevalence (2.74%). On the other hand, the new B-12 cutoff had a better-balanced sensitivity/specificity, as reflected by the higher Youden’s index (0.69), compared with the traditional cutoffs (other than B-12 <223, which is nearly identical to <220 pmol/L). Thus, in the absence of MMA, B-12 <148 pmol/L may be best suited in a presumably healthy population, whereas a higher threshold, such as B-12 <220 pmol/L, may be preferable to screen a high-risk group (e.g., older persons or populations with a higher prevalence of B-12 deficiency).

Although we were interested to assess how commonly used traditional cutoffs fared in terms of diagnostic accuracy compared with 3cB-12 ≤−0.5 indicating inadequate vitamin B-12 status, it is not surprising that we found the lowest diagnostic accuracy (Youden’s index) for traditional cutoffs typically used to assess vitamin B-12 deficiency (rather than insufficiency), such as B-12 <148 pmol/L (0.41) and B-12 <126 pmol/L (0.30). These cutoffs have very high specificity (>99%) but they suffer from poor sensitivity (41.9% and 30.6%, respectively). It is important to keep in mind that our analysis did not focus on vitamin B-12 deficiency because the prevalence in our sample is too low for a meaningful analysis.

Our subanalysis in all adults regardless of their renal function produced consistent results to our main analysis limited to adults with normal renal function: MMA had again the highest predictive power and the new optimum cutoff had the highest diagnostic accuracy as assessed by Youden’s index compared with all other markers and cutoffs. As expected, the new optimum cutoffs in our subanalysis were slightly different from those in our main analysis for B-12 (<240 compared with <220 pmol/L) and MMA (>259 compared with >229 nmol/L) but interestingly not for tHcy (>10.4 compared with >10.4 μmol/L) and the 3cB-12 reference prevalence was slightly higher (3.23% compared with 2.74%), reflecting typically increased biomarker concentrations in the presence of renal impairment.

The strengths of our paper are the nationally representative nature of the data and the large sample size from 3 cycles (6 y) of NHANES that allowed us to use 2 cycles of the dataset to derive new optimum cutoffs and a third cycle to independently assess their diagnostic accuracy. Limiting our main analysis to apparently healthy adults with normal renal function reduced the impact of an important confounding factor. Furthermore, conducting a full subanalysis in all adults regardless of their renal function allowed us to assess the consistency of findings across both analyses, providing further support for our conclusions. Lastly, the reporting of the AUC and Youden’s index provided both threshold-independent (discrimination) and threshold-dependent (clinical utility) performance metrics. Our main limitation compared with some of the other reports assessing the diagnostic accuracy of conventional markers relative to cB-12 is that we were unable to include holoTC and do not know how that may have changed our findings. Moreover, the reported optimum cutoffs may not be applicable to all age groups as they were derived for US adults. An earlier analysis of MMA in NHANES 2011–2014 showed increasing concentrations with age, independent of renal function and vitamin B-12 status [20]. Although a sex- and/or age-stratified analysis like the one performed by Campos et al. [15] was beyond the scope of our study, an evaluation of subgroup-specific cutoffs and biomarker performance represents an important area for future research. Lastly, our study is subject to all limitations that apply to cross-sectional data.

When interpreting findings in the context of NHANES, it is important to consider potential differences in analytic methods across survey cycles as well as the availability of biomarker data. MMA has first been measured using GC-MS (1999−2004) and in later cycles using liquid chromatography-tandem mass spectrometry. A previous evaluation has demonstrated excellent agreement and minimal bias between these 2 methods (e.g., Pearson *r* = 0.99), supporting the equivalent use of MMA data across cycles for population-level analyses [38]. Although more recent NHANES cycles (2011−2014) include serum vitamin B-12 and MMA data, they do not include tHcy data, which limits the direct application of multimarker approaches such as those evaluated here.

In summary, MMA showed the strongest ability to identify inadequate vitamin B-12 status defined by 3cB-12 ≤−0.5. The optimal MMA cutoff selection depends on the intended application. In a generally healthy population with low (<5%) prevalence of inadequate vitamin B-12 status, MMA >376 nmol/L may minimize false positives and provide prevalence estimates closely aligned with 3cB-12 estimates. In settings where maximizing case detection is a priority, such as screening cases with higher risk or populations with a higher prevalence of B-12 deficiency, MMA >229 nmol/L provides the overall best combination of sensitivity and specificity.

## Author contributions

The authors’ responsibilities were as follows – EMM, CMP, MRS: designed the research project; EMM, MRS, CMP: conducted the research; MRS: analyzed the data; EMM: wrote the initial draft of the paper; CMP: had primary responsibility for final content and EMM, MRS, CMP: read and approved the final manuscript.

## Data availability

The NHANES data are publicly available at https://wwwn.cdc.gov/nchs/nhanes/default.aspx.

## Declaration of Generative AI and AI-Assisted Technologies in the Writing Process

No generative AI and AI-assisted technologies were used in preparing the manuscript. An internal general-purpose assistant (CDC ChatGPT 5.3, OpenAI) was used during revisions to provide suggested improvements for readability and language of the work. After using this tool/service, the authors reviewed and edited the content as needed and take full responsibility for the content of the publication.

## Funding

The authors reported no funding received for this study.

## Conflict of interest

The authors report no conflicts of interest.
