# Supplementary material for: The Diagnostic Accuracy of Methylmalonic Acid to Detect Inadequate Vitamin B-12 Status Relative to 3cB-12 Was Higher Compared with That of Total Cobalamin and Total Homocysteine in a Cross-Sectional Survey of Apparently Healthy US Adults
Source: Curr Dev Nutr. 2026 Apr 22;10(6):107704. doi: 10.1016/j.cdnut.2026.107704 (PMC13200092; doi:10.1016/j.cdnut.2026.107704)
Supplement: multimedia component 1 [file mmc1.docx]

The diagnostic accuracy of methylmalonic acid to detect inadequate vitamin B_12_ status relative to 3cB_12_ was higher compared to that of total cobalamin and total homocysteine in a cross-sectional survey of apparently healthy U.S. adults

First author: Mineva EM

**Supplementary Table 1.** Area under the curve (AUC) of the receiver operating characteristic (ROC) to detect inadequate (low or transitional) vitamin B_12_ status (3cB_12_ ≤-0.5) for various models of single or combined conventional biomarkers in U.S. adults aged 20 y and older, NHANES 1999−2002^1^

| **Biomarker models** | **Weighted AUC (95% CI)^2^** |
| --- | --- |
| *Single Biomarkers* |  |
| B_12_ | 0.906^a^ (0.876, 0.935) |
| MMA | 0.966^b^ (0.951, 0.980) |
| tHcy | 0.934^a^ (0.918, 0.949) |
| *Multiple Biomarkers* |  |
| B_12_, MMA | 0.983^c, d^ (0.972, 0.992) |
| B_12_, tHcy | 0.962^b, d^ (0.947, 0.978) |
| MMA, tHcy | 0.990^c^ (0.987, 0.993) |
| MMA, tHcy, B_12_ | 0.997^e^ (0.996, 0.998) |

Abbreviations: B_12_, serum vitamin B_12_; CI, confidence interval; MMA, plasma methylmalonic acid; tHcy, plasma total homocysteine.

^1^ Pregnant and lactating women were excluded; only participants ≥20 y with complete biomarker data were included. Sample size was *n*=8,255 participants of whom 231 were categorized as having inadequate vitamin B_12_ status.

^2^ Groups with different superscript letters are significantly different from each other based on pairwise testing (*p* <0.05). Bootstrap 95% confidence intervals.

**Supplementary Table 2.** Receiver operating characteristic (ROC) derived optimum cutoffs and corresponding diagnostic performance to detect inadequate (low or transitional) vitamin B_12_ status (3cB_12_ ≤-0.5) based on single conventional biomarkers in U.S. adults aged 20 y and older, NHANES 1999−2002^1^

| **Biomarker** | **Optimum cutoff ^2^**  **(95% CI)** | **Sensitivity, % (95% CI)^3^** | **Specificity, %**  **(95% CI)^3^** | **Youden’s**  **index**^4^ |
| --- | --- | --- | --- | --- |
| B_12_ | 240 (204, 252) | 87.3 (80.7, 91.8) | 81.8 (80.8, 82.8) | 0.69 |
| MMA | 259 (230, 270) | 87.2 (78.1, 92.8) | 94.2 (93.6, 94.8) | 0.81 |
| tHcy | 10.4 (10.3, 12.4) | 90.5 (86.2, 93.6) | 80.6 (79.3, 81.8) | 0.71 |

Abbreviations: B_12_, serum vitamin B_12_; CI, confidence interval; MMA, plasma methylmalonic acid; tHcy, plasma total homocysteine.

^1^ Pregnant and lactating women were excluded; only participants ≥20 y with complete biomarker data were included. Sample size was *n*=8,255 participants of whom 231 were categorized as having inadequate vitamin B_12_ status.

^2^ Optimum cutoff points for the conventional markers correspond to the value with the minimum distance to the (0,1) point on the ROC plot (optimized sensitivity and specificity). Confidence intervals for the optimal cutoff points were derived from bootstrap 95% confidence intervals.

^3^ Standard errors were estimated using Taylor series linearization. Confidence intervals for sensitivity and specificity were calculated using a logit transformation.

^4^ Youden’s index was calculated as sensitivity + specificity - 1.

**Supplementary Table 3.** Diagnostic performance and weighted apparent and adjusted prevalence for optimum and traditional cutoffs for U.S. adults aged 20 y and older, NHANES 2003–2004^1^

| **Biomarker categories (reference for cutoffs)** | **Sensitivity, %**  **(95% CI)^2^** | **Specificity, %**  **(95% CI)^2^** | **Youden’s index^3^** | **Apparent**  **prevalence, %**^4^  **(95% CI)^2^** | **Adjusted prevalence, %**^4^  **(95% CI)^2^** |
| --- | --- | --- | --- | --- | --- |
| 3cB_12_^5^ <-0.5 (12) | 100 | 100 | – | 3.23 (2.39, 4.34) | 3.23 (2.39, 4.34) |
| **Optimum cutoffs** |  |  |  |  |  |
| B_12_ <240 pmol/L | 88.6 (81.1, 93.3) | 83.1 (80.1, 85.7) | 0.72 | 19.3 (16.4, 22.5) | 1.50 (0.06, 26.8) |
| MMA >259 nmol/L | 84.4 (76.9, 89.7) | 94.2 (92.6, 95.5) | 0.79 | 8.29 (6.65, 10.3) | 3.10 (1.43, 6.58) |
| tHcy >10.4 µmol/L | 90.6 (79.8, 95.9) | 78.1 (74.9, 80.9) | 0.69 | 24.2 (21.1, 27.5) | 6.66 (3.19, 13.5) |
| **Traditional cutoffs** |  |  |  |  |  |
| B_12_ <148 pmol/L (29) | 36.8 (30.0, 44.2) | 99.1 (98.6, 99.5) | 0.36 | 2.02 (1.44, 2.85) | 1.66 (0.48, 5.56) |
| B_12_ <223 pmol/L (29) | 85.2 (77.0, 90.8) | 87.5 (84.1, 90.3) | 0.73 | 14.8 (11.8, 18.5) | 1.52 (0.05, 32.5) |
| B_12_ <126 pmol/L (30) | 27.8 (19.3, 38.2) | 99.6 (99.2, 99.8) | 0.27 | 1.29 (0.90, 1.85) | 3.11 (1.47, 6.46) |
| B_12_ <287 pmol/L (30) | 93.8 (88.1, 96.8) | 67.7 (64.4, 70.8) | 0.61 | 34.3 (31.0, 37.8) | 2.90 (0.36, 19.9) |
| MMA >376 nmol/L (31) | 60.1 (48.7, 70.5) | 98.8 (98.2, 99.1) | 0.59 | 3.13 (2.51, 3.91) | 2.80 (1.72, 4.51) |
| MMA >271 nmol/L (31) | 82.4 (74.1, 88.5) | 95.0 (93.5, 96.1) | 0.77 | 7.55 (6.06, 9.35) | 3.81 (2.10, 6.81) |
| MMA > age-specific cutoff (20) | 76.7 (65.5, 85.1) | 96.2 (95.1, 97.0) | 0.73 | 6.17 (4.97, 7.64) | 2.79 (1.41, 5.47) |
| tHcy >13 µmol/L (32) | 74.9 (65.7, 82.3) | 92.7 (91.3, 93.9) | 0.68 | 9.49 (7.94, 11.3) | 5.00 (2.84, 8.67) |

Abbreviations: B_12_, serum vitamin B_12_; CI, confidence interval; MMA, plasma methylmalonic acid; tHcy, plasma total homocysteine.

^1^ Pregnant and lactating women were excluded; only participants ≥20 and complete biomarker data were included. Sample size was *n*=4,086 participants, of whom 136 were categorized as having inadequate vitamin B_12_ status.

^2^ Standard errors were estimated using Taylor series linearization. Confidence intervals for sensitivity, specificity, and prevalence were calculated using a logit transformation.

^3^ Youden’s index was calculated as sensitivity + specificity - 1.

^4^Apparent prevalence was adjusted using the sensitivity and specificity of the traditional cutoffs estimated from NHANES 1999–2002.

^5^ 3cB_12_ was considered the gold standard with 100% sensitivity and 100% specificity.

**Supplementary Figure 1.** Number of U.S. persons who were screened, interviewed, examined, for whom biomarker concentrations were measured, and who were excluded due to age, pregnancy/lactation status, missing biomarker data, or renal function NHANES 1999**–**2004*

Abbreviations: B_12_, vitamin B_12_; MMA, methylmalonic acid; P/L, pregnancy/lactation status; tHcy, total homocysteine.

* Sample size for the calculation of combined vitamin B_12_ indicator (cB_12_)

Adequate B_12_ status defined as 3cB_12_ >-0.5; inadequate B_12_ status defined as 3cB_12_ ≤-0.5.

Abnormal renal function defined as GFR <60; normal renal function defined as GFR ≥60.

Screened

Interviewed

Examined

**1999–2002**

25,316

21,004

19,759

**2003–2004**

12,761

10,122

9643

Serum B_12_ measured

Plasma MMA measured

Plasma tHcy measured

All 3 biomarkers measured

*Total exclusions among subjects with all 3 biomarkers measured:*

<20 y of age

P/L women ≥20 y

not P/L ≥20 y with abnormal renal function

**Analytic Sample with normal renal function**

**Adequate B_12_status**

**Inadequate B_12_ status**

15,914

16,048

16,071

15,853

*8,384*

7,012

586

786

**7,469**

**7,312**

**157**

8,267

7,544

7,888

7,496

*3,855*

3,184

226

445

**3,641**

**3543**

**98**

**Training Data**

**Validation Data**
